# Duodenal–Jejunal Bypass Restores Sweet Taste Receptor-Mediated Glucose Sensing and Absorption in Diabetic Rats

**DOI:** 10.1155/2024/5544296

**Published:** 2024-09-04

**Authors:** Sipeng Sun, Anping Wang, Rongguan Kou, Hantao Xue, Xiangyu Zhao, Ben Yang, Mengqi Shi, Yubing Wang, Qingtao Yan, Meihua Qu, Yi Wang, Zhiqin Gao

**Affiliations:** ^1^ School of Life Science and Technology Shandong Second Medical University, Weifang 261021, China; ^2^ Department of Pediatric Surgery Weifang People's Hospital The First Affiliated Hospital of Shandong Second Medical University, Weifang 261021, China; ^3^ Translational Medical Center Weifang Second People's Hospital, Weifang 261021, China

**Keywords:** DJB, GLP-1, GLUT2, lactisole, T1R2

## Abstract

**Aim:** The aim of the study is to identify the regulatory role of intestinal sweet taste receptors (STRs) and glucose transporters (SGLT1, GLUT2) and gut peptide secretion in duodenal–jejunal bypass (DJB)–ameliorated glycemic control in Type 2 diabetes.

**Materials and Methods:** DJB and sham surgeries were performed in streptozotocin-induced diabetic male rats. The blood GLP-1 and GLP-2 levels were evaluated under feeding and fasting conditions. The expression of STRs (T1R2, T1R3), sweet taste signaling effector (G*α*-gustducin), SGLT1, and GLUT2 was detected in the intestinal alimentary limb (A limb), biliopancreatic limb (BP limb), and common limb (C limb). The effects of STR inhibition on glucose control were measured with lactisole.

**Results:** Glucose tolerance was improved in DJB-operated rats compared with the sham group, similar to that of normal control rats, without significant differences in food intake and body weight. The plasma GLP-1 levels of DJB rats were increased under diet-fed condition, and GLP-2 levels were increased after fasting. The villus height and crypt depth were significantly increased in the A limb of DJB-operated rats. In addition, GLP-1 expression was restored in enterocytes. The expression of T1R2, G*α*-gustducin, and SGLT1 was elevated in the A limb after DJB, while GLUT2 was downregulated in the A, BP, and C limbs. The localization of GLUT2 was normalized in the three intestinal limbs after DJB. However, the beneficial effects of DJB on glucose control were abolished in the presence of lactisole in vivo.

**Conclusion:** DJB ameliorates glycemic control probably by restoring STR-mediated glucose sensing and absorption with the responses of GLP-1 and GLP-2 to carbohydrate.

## 1. Introduction

Gastrointestinal bypass surgery, a type of metabolic surgery, has been performed to improve metabolic homeostasis, including glycemic control, in both humans and rodents with diabetes. Sleeve gastrectomy (SG) and Roux-en-Y gastric bypass (RYGB) are commonly performed in obese and diabetic individuals to effectively achieve weight loss and glucose homeostasis improvement [1, 2]. SG with duodenal–jejunal bypass (DJB) surgery was later developed as an advanced clinical therapy in Asians with obesity and/or diabetes. It has been reported that blood glucose was decreased earlier in obese rats after SG-DJB than in the SG group. DJB was originally designed to investigate the underlying mechanisms of RYGB-induced antidiabetic effects but has been found to potentiate metabolic benefits in obese and diabetic models [3–5]. However, the mechanisms underlying DJB-improved glycemic control remain to be understood.

It is known that ingested nutrients, including sugars, encounter the gastrointestinal tract first after eating. The small intestine is responsible for dietary nutrient absorption from the gut lumen to the blood circulation system through corresponding receptors and transporters on enterocytes and enteroendocrine cells [6]. Among them, glucose is sensed by sweet taste receptors (STRs) and T1R2/T1R3 heterodimers on enteroendocrine L cells [7] and is primarily transported by sodium-glucose cotransporter 1 (SGLT1) on the apical membrane of enterocytes and glucose transporter 2 (GLUT2) on the basolateral membrane of enterocytes [6]. Many studies have revealed that SGLT1-mediated glucose uptake triggers the release of glucagon-like peptide-1 (GLP-1) from L cells, which regulates insulin secretion [6, 8]. SGLT1 and GLP-1 are also the most studied due to their changes involved in RYGB- and DJB-improved glucose homeostasis [9, 10]. However, the role of glucagon-like peptide-2 (GLP-2), which is coreleased with GLP-1 from L cells, has rarely been explored in DJB surgery. It has been reported that STR-mediated glucose sensing could potentiate glucose absorption by regulating glucose transport under the coordination of GLP-1 and GLP-2 in the murine gut [7]. In addition, glucose and artificial sweetener-induced GLP-2 secretion was found to upregulate the expression levels of SGLT1 in the proximal small intestine [11].

In this study, we focused on the changes of intestine tissue in glucose sensing and absorption before and after metabolic surgery, so we chose a DJB surgery. DJB surgery only reconstructs the small intestine without changes in gastric tissue. Our previous studies demonstrated that DJB ameliorated glucose intolerance in nonobese diabetic rats and improved glucose uptake in the brain [4] and glucose metabolism under fasting condition [12]. While DJB remolds the small intestine initially, so what changes occur in the reconstructed intestinal duodenum and jejunum before and after DJB? This study therefore investigated the alterations in both GLP-1 and GLP-2 levels after DJB operation in a Type 2 diabetic rat model under feeding and fasting conditions and examined the changes in STR signals (T1R2/T1R3/G*α*-gustducin [G protein alpha gustducin]) and glucose transporters (SGLT1/GLUT2) in the DJB-remodelled duodenum and jejunum. Furthermore, the STR inhibitor, lactisole, which has been found to inhibit the secretion of GLP-1 under high glucose condition in STC-1 cells, was first used to identify the role of STR in Type 2 diabetic rats after DJB.

## 2. Materials and Methods

### 2.1. Animals and Glucose Tolerance Test

Eight-week-old male Wistar rats were kept in captivity under SPF conditions (ambient temperature 22°C–24°C, 12-h light/dark cycle). Rats could drink and eat freely. The male rats were randomly divided into three groups: the control group (control group, *n* = 6–7), DJB intervention group (T2D-DJB, *n* = 5–6), and sham operation group (T2D sham operation group, *n* = 5–6). The nonobese Type 2 diabetic rat model was induced by a fat diet (25.4% fat, 57.4% carbohydrates, and 17.2% protein) (Shanghai Fanbo Biotechnology Co., Ltd. Company, 4th Floor, Building 3455, Shendu Road, Shanghai, China) for 8 weeks and received a low-dose streptozotocin (STZ, 28 mg/kg; Sigma, American) injection. All procedures involving animals were reviewed and approved by the Animal Ethics Committee of Weifang Medical University (no. 2020SDL074). An oral glucose tolerance test (OGTT) with 2 g/kg glucose was performed 1 week preoperatively and 6 weeks postoperatively after 12 h of fasting in rats. Homeostatic model assessment-insulin resistance (HOMA-IR) was calculated according to the following formula: HOMA − IR = fasting insulin (mIU/L) × fasting glucose (mmol/L)/22.5. ITT (1 U/kg) was also performed 6 weeks postoperatively after 12 h of fasting in rats. The blood glucose level was measured at 0, 30, 60, 90, and 120 min.

### 2.2. DJB Surgery

DJB and sham operation were performed under local anesthesia with lidocaine hydrochloride as described previously [4, 12]. Briefly, the animals were fasted overnight prior to surgery and had free access to water. The rats were then injected subcutaneously with 0.5% lidocaine hydrochloride (0.3 mL/500 g BW) for local anesthesia, and the abdominal cavity of the rats was cut along the midline of the abdomen. Then, absorbable surgical sutures were used to tie the pyloric part of the stomach to the junction of the duodenum, the pyloric end close to the stomach was cut off, and sterile cotton balls were used to stop bleeding. A sterile syringe was used to connect the scalp needle hose, and the food residue in the stomach was gently extracted. Find the Treitz ligament along the duodenum, and cut the jejunum 5 cm, divide the small intestine into proximal jejunum and distal jejunum, and lift the distal jejunum up and suture it with the lower gastric stump. The anastomosis was sutured with the proximal jejunum by cutting the opening 10 cm downwards. After the operation, the small animals were placed in the incubator to recover their temperature.

### 2.3. Sample Collection

At the 7th week after the operation, the rats were killed under normal feeding conditions, and the intestinal tissues were preserved. In addition, cardiac blood was collected and stored at −80°C for further study. The whole intestinal limb of rats was taken and washed with 0.9% normal saline. DJB rats were divided into biliopancreatic limb (BP limb), alimentary limb (A limb), and common limb (C limb) according to the intestinal status after surgery. Control and T2DM sham rats were divided into duodenum and 5 cm jejunum according to BP segment, Segment A was 10 cm jejunum, and Segment C was the remaining small intestine segment. After removing the mesentery, part of the intestine was placed in 4% paraformaldehyde at 4°C, and the other part was stored at −80°C. The rat small intestine was flushed with ice-cold PBS and cut open lengthwise to expose the side of the intestinal cavity. A slide was used to gently scrape off the villi of the small intestine, and the villi were placed in RIPA buffer for protein expression and TRIzol for mRNA expression.

### 2.4. Enzyme-Linked Immunosorbent Assay

The collected blood samples of rats were centrifuged at 3500 rpm at 4°C for 10 min; the upper serum was collected. The plasma total insulin, GLP-1, and GLP-2 were measured by enzyme-linked immunosorbent assay (cat# CEA448Ra, cat# CEA804Ra, and cat# CED059Ra, Cloud-Clone Corp., formerly Uscn Life Science Inc., Wuhan, China) according to the manufacturer's instructions. Briefly, 100 *μ*L of the collected plasma was added in the microplate wells within ELISA kit and incubated for 2 h. Discard the liquid, and add 100 *μ*L biotinylated antibody working solution in each well for 1 h. Discard the liquid, and wash for three times. Then, add 100 *μ*L horseradish peroxidase-labeled avidin working solution for 1 h, followed by 90 *μ*L substrate solution for 15–25 min, and finally add 50 *μ*L stop solution to stop the reaction. The optical density of each well was measured at 450 nm in a microplate reader, and the hormone concentration of each sample was calculated according to the standard curve.

### 2.5. RNA Sequencing and Analysis

Intestinal A limbs were isolated from DJB-operated rats and sham-operated rats for transcriptome RNA sequencing (Shanghai GeneChem Co., Ltd.). The role of glucose perception- and absorption-related signals in the digestive tract after DJB was analyzed. The downstream analysis used volcano, Gene Ontology (GO), and GSEA enrichment.

### 2.6. Quantitative Real-Time PCR

Total RNA of small intestinal villi from different intestinal segments was extracted and reverse-transcribed into cDNA by a reverse transcription kit (cat# FSQ-101, Toyobo, Osaka, Japan). SYBR Green Real-Time PCR Master Mix (cat# MF788-01, Mei5bio, Beijing, China) was used in the study. Primer sequences are shown in Table S1 (Thermo Fisher Scientific). The relative mRNA expression of the target genes was analyzed using 2^−ΔΔCT^.

### 2.7. Western Blot Analysis

Scraped intestinal mucosa of the A limb, BP limb, and C limb was prepared as previously described. The intestinal villus sample protein was subjected to 10% SDS–PAGE, incubated with primary anti-T1R2 (1:1000; cat# PA5-99935, Invitrogen), anti-T1R3 (1:1000; cat# NB100-98792, NOVUS), anti-GNAT3/G*α*-gust (1:1000; cat# bs-6149R, Bioss), anti-SGLT1 (1:500; cat# 5042, Cell Signaling Technology), and anti-GLUT2 (1:1000; cat# bs-10379R, Bioss), and then incubated with the corresponding secondary antibodies (Cell Signaling Technology).

### 2.8. Histological Observation

HE staining was used to check the tissue morphology. The small intestines of rats in the three groups were collected and fixed with 4% paraformaldehyde. Fixed tissues were embedded in paraffin. Embedded tissue sections (5 *μ*m) were dewaxed and stained with hematoxylin for 5 min. After identification with hydrochloric acid alcohol and washing with tap water, the slices were dyed with eosin for 30 min, dehydrated in anhydrous ethanol of different concentrations, and treated with xylene, and then, the slices were transparently sealed with neutral balsam. The villus height and recess depth of the small intestine were measured by ImageJ software.

### 2.9. Immunofluorescence

Intestinal segments from the dissected rats were fixed in 4% paraformaldehyde and embedded in paraffin for 48 h. Five-micron-thick slices were prepared with a paraffin slicer and collected on the slide immediately. The embedded tissue sections (5 *μ*m) were dewaxed and incubated with primary antibodies, such as anti-T1R2 (1:100), anti-T1R3 (1:100), anti-GLUT2 (1:200; cat# 66889-1-Ig, Proteintech, Wuhan, China), and sucrase (1:200; cat# PA5-95609, Invitrogen), at 4°C and washed with PBS. The slide was incubated with a fluorescent conjugated secondary antibody (Invitrogen-Alexa) for 1 h. Then, the nuclei were stained with 4-amino-6-diamino-2-9-phenyl indole (DAPI, Life Technologies) for 5 min. The fluorescence signal was examined by confocal inverted microscopy.

### 2.10. Effect of Lactisole on Glucose Tolerance

Four weeks after the operation, the rats in the three groups were fasted overnight and then orally administered 2 g/kg glucose or 2 g/kg glucose + 10 mg/kg lactisole (cat# GC44024, GLPBIO, United States). The blood glucose of rats was measured at 0, 15, 30, 60, 90, and 120 min after gastric gavage. The area under the curve (AUC) was calculated according to the OGTT results.

### 2.11. Statistical Analysis

The data are shown as the mean ± SD using GraphPad 8 software. A *t* test was used for the change in a factor before. ANOVA and Tukey's test were used for the comparison of multiple groups, and the trapezoidal accumulation method was used for the calculation of the AUC. *p* < 0.05 was set as significant.

## 3. Results

### 3.1. Glucose Tolerance Is Improved in Type 2 Diabetic Rats After DJB With the Alteration of Blood Hormones

**Figure 1:**
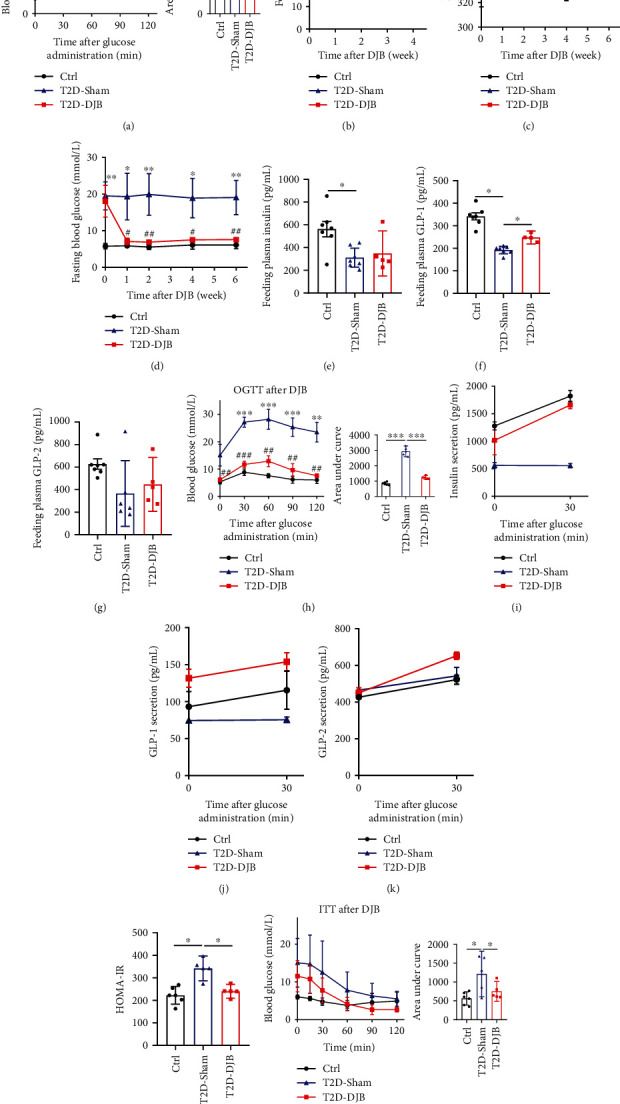
Effects of duodenal–jejunal bypass on body weight, food intake, and glucose homeostasis in diabetic rats. Oral glucose tolerance test (OGTT) and area under curve for (a) OGTT before surgery as indicated. (b) Body weight, (c) food intake, and (d) fasting blood glucose before and after surgery. (e) Feeding plasma insulin, (f) GLP-1 (f), and (g) GLP-2 concentrations in chow diet-fed rats for 5 weeks after DJB or sham operation. (h) OGTT with area under curve, (i) the secretion of insulin, (j) GLP-1, (k) GLP-2 during OGTT, (l) HOMA-IR, and (m) ITT were examined 6 weeks postsurgery. Ctrl group (black, *n* = 6), T2D-Sham group (blue, *n* = 5–6), T2D-DJB group (red, *n* = 5–6). Data are presented as the mean ± SD. ^∗^*p* < 0.05, ^∗∗^*p* < 0.01, and ^∗∗∗^*p* < 0.001 versus the Ctrl group; ^#^*p* < 0.05, ^##^*p* < 0.01, and ^###^*p* < 0.001 versus the T2D-Sham group.

To determine the effects of DJB on glycemic control, DJB and sham surgeries were performed on STZ-induced nonobese diabetic Wistar male rats. The preoperative OGTT results showed that T2D-DJB and T2D-Sham rats displayed impaired glucose tolerance compared with Ctrl rats, while there was no difference between the T2D-DJB and T2D-Sham groups (Figure 1(a)). After surgery, although DJB-operated rats did not exhibit significantly lower food intake and body weight than sham-operated rats (Figures 1(b) and 1(c)), DJB rats displayed a substantial decrease in fasting blood glucose notably 1 week after DJB operation (Figure 1(d)). We also examined the effects of DJB on insulin, GLP-1, and GLP-2 hormones mediating glucose homeostasis. The plasma insulin levels of T2D-Sham rats were significantly lower than those of Ctrl rats in a chow diet-fed state 5 weeks postsurgery (Figure 1(e)). Feeding plasma GLP-1 levels were also significantly lower in sham-operated rats (Figure 1(f)). However, a marked elevation was observed in GLP-1 levels in DJB-operated rats compared with the sham group, while the postoperative circulating GLP-2 levels did not increase significantly after DJB under chow diet-feeding condition (Figure 1(g)).

Consistent with the decrease in fasting glucose levels, the DJB group showed a significant improvement in glucose intolerance (Figure 1(h)) with increased responses of insulin, GLP-1, and GLP-2 secretion (Figures 1(i), 1(j), and 1(k)) during OGTT, as well as improved insulin intolerance 6 weeks postsurgery (Figures 1(l) and 1(m)).

### 3.2. DJB Induced Adaptive Changes in Villus Height and Crypt Depth of Intestinal Segments

**Figure 2:**
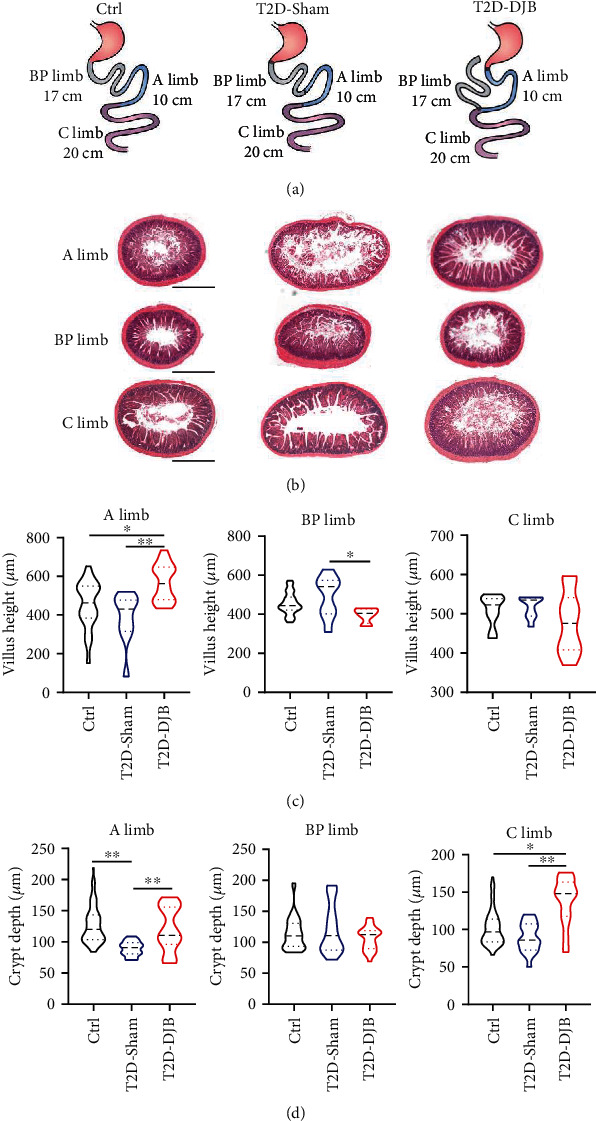
Changes in villus height and crypt depth in the alimentary limb, biliopancreatic limb, and common limb after DJB. (a) Schematic diagram of DJB and sham operation. (b) Cross-sections of intestinal segments of Ctrl, T2D-Sham, and T2D-DJB rats 6 weeks postsurgery (HE staining). (c) Villus height and (d) crypt depth in the A limb, BP limb, and C limb of Ctrl, T2D-Sham, and T2D-DJB rats. Scale bar, 1 mm. Data are presented as the mean ± SD. ^∗^*p* < 0.05; ^∗∗^*p* < 0.01.

We then compared the villus height and crypt depth of intestinal segments in rats subjected to DJB or sham surgery as well as Ctrl rats. DJB rats exhibited obvious structural adaptation in the A limb, BP limb, and C limb (Figure 2(a)). As shown in Figure 2(b), the intestinal mucosa of the A limb was markedly hyperplastic after the DJB operation. The villus height of the A limb was dramatically increased in DJB rats compared to the Ctrl and sham groups (Figure 2(c)), and the crypt depth of the A limb was decreased in sham rats but also increased after DJB operation (Figure 2(d)). The villus height and crypt depth were decreased in the BP limb of DJB rats compared with sham rats (Figures 2(c) and 2(d)), whereas no significant changes in villus height were observed in the C limb (Figure 2(c)). However, the crypt depth of the C limb was greater in DJB rats than in sham and Ctrl rats (Figure 2(d)).

### 3.3. Effects of DJB on Glucose Sensor and Transporter Expression in the A Limb, BP Limb, and C Limb

**Figure 3:**
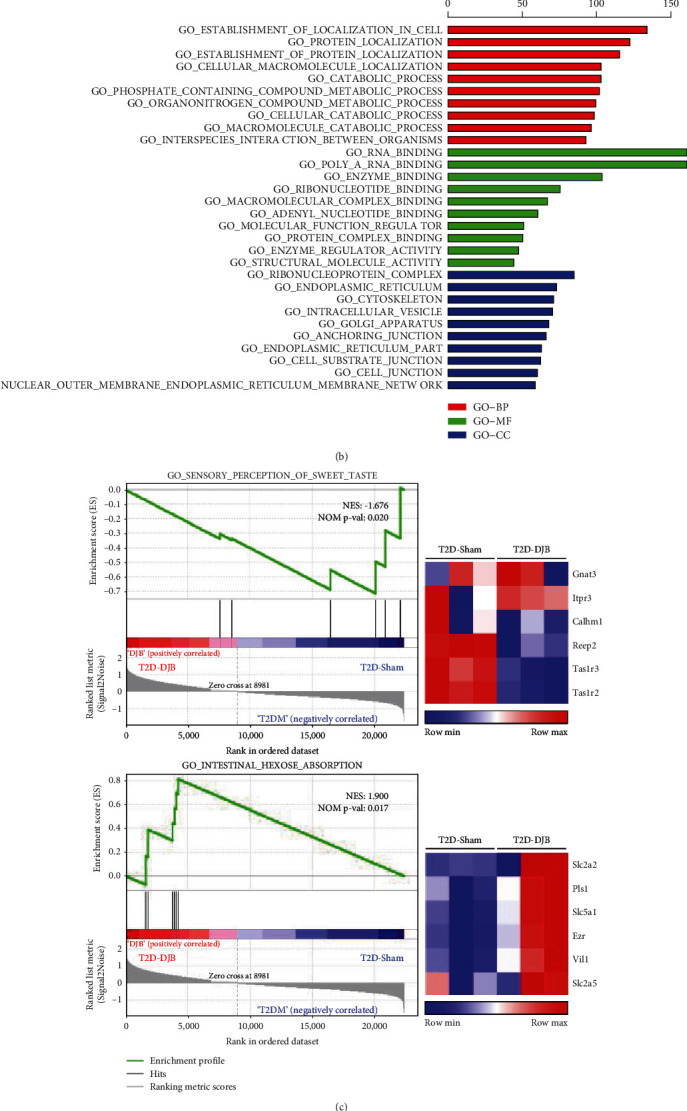
Transcriptomic analysis revealed that glucose sensing and absorption-related signaling played important roles in the alimentary limb after DJB. (a) Volcano plot of differentially expressed genes (DEGs) between T2D-Sham and T2D-DJB in the alimentary limb using the following thresholding criteria: fold change > 2 and false discovery rate (FDR) *p* value < 0.05. (b) Gene Ontology (GO) analysis delineated the enrichment of differentially expressed genes in three major categories, named biological process (BP), molecular function (MF), and cellular components (CC). (c) GSEA showed the sensory perception of the sweet taste–associated gene set and intestinal hexose absorption-associated gene set. (d) Expression of T1R2 and T1R3 in the alimentary limb. Immunofluorescence analysis with T1R2 (red), T1R3 (green), and DAPI (blue) in sections of the rat alimentary limb. The arrows (white) denote the colocalization of T1R2 and T1R3 in the small intestine. NES = Normalized Enrichment Score. Positive and negative values of NES indicated up- and downregulated gene expression patterns, respectively. Heatmap showing the relative RNA expression of genes included in specific gene sets.

The improved glucose control, gut peptide secretion, and adaptive responses in intestinal mucosa resulted from the changes in small intestinal segments after DJB. To identify the gene expression changes in the small intestine, we conducted RNA sequencing on the A limb from sham and DJB rats. The results showed that 2104 genes were upregulated and 439 genes were downregulated in the adaptive A limb after DJB (Figure 3(a)). GO enrichment analysis showed that the differentially expressed genes were specifically involved in protein localization and catabolic processes in the A limb after DJB (Figure 3(b)). From these, we analyzed genes that encoded nutrition-sensing member proteins and transporters and found that the expression of genes involved in the sensory perception of sweet taste was decreased, while the expression of genes involved in intestinal hexose absorption was enhanced in the A limb after DJB compared with that of the T2D-Sham group (Figure 3(c)). From the gene sets, we selected Tas1r2 (T1r2), Tas1r3 (T1r3), Gnat3 (G*α*-gustducin), Slc5a1 (Sglt1), and Slc2a2 (Glut2), which play important roles in intestinal glucose sensing and absorption.

**Figure 4:**
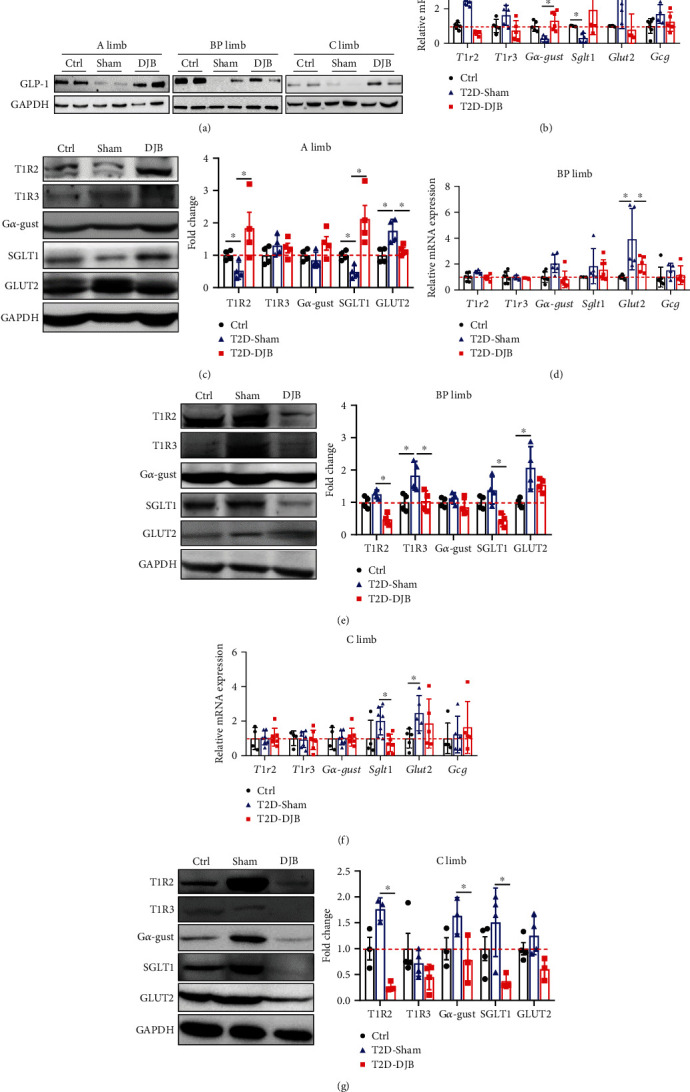
Expression levels of glucose sensors and transporters were altered in the A limb, BP limb, and C limb after DJB. The expression levels of (a) GLP-1, T1R2, T1R3, G*α*-gust, SGLT1, and GLUT2 were detected by real-time PCR and western blot, respectively, in the (b, c) A limb, (d, e) BP limb, and (f, g) C limb of T2D-DJB (DJB) rats and corresponding limbs of Ctrl and T2D-Sham (sham) rats. Fold change was calculated based on the Ctrl limbs and normalized to GAPDH. Data are presented as the mean ± SD. ^∗^*p* < 0.05.

We further validated the expression of GLP-1, glucose sensors, and glucose transport genes in the altered intestinal mucosa by real-time quantitative PCR and western blotting. The protein levels of GLP-1 in the intestinal epithelial cells were reduced in the sham groups but restored after DJB (Figure 4(a)), although there was no significant difference in gcg mRNA expression. T1r2 mRNA expression in the A limb was decreased after DJB, whereas it was increased in sham rats (Figure 4(b)). Interestingly, the changes in the protein levels of T1R2 were contrary to those in the mRNA levels in the A limb (Figure 4(c)). Although T1R2 and T1R3 were coexpressed in the A limb (Figure 3(d)), T1R3 expression levels did not differ significantly (Figures 4(b) and 4(c)). However, the expression of the sweet taste signaling effector G*α*-gustducin (G*α*-gust) tended to increase after DJB (Figures 4(b) and 4(c)). The expression levels of the main intestinal glucose transporter, SGLT1, were also markedly increased in the hypertrophic A limb of DJB rats (Figures 4(b) and 4(c)). In contrast, the expression levels of GLUT2 were lower in the A limb of DJB rats than in that of sham rats (Figures 4(b) and 4(c)). In addition, we detected the expression changes of these genes in the BP limb and C limb. The mRNA expression of T1r2 was increased in the BP limb of sham rats compared to that of Ctrl rats but decreased in the BP limb of DJB rats compared with sham rats (Figure 4(d)), consistent with the western blot results (Figure 4(e)). There was no difference in T1r3 gene expression in the BP limb among the three groups (Figure 4(d)), but the western blot data showed that the protein levels of T1R3 were higher in sham rats than in Ctrl rats and significantly decreased after DJB operation (Figure 4(e)). We found that the changes in G*α*-gust expression were similar to those of T1R2 in BP limbs after surgery (Figures 4(d) and 4(e)). The expression of SGLT1 and GLUT2 appeared to be increased in the BP limb of sham rats but decreased in DJB rats at both the mRNA and protein levels (Figures 4(d) and 4(e)). The expression changes of these genes in the C limb were similarly decreased, especially T1R2 and SGLT1 in DJB-operated rats compared to sham-operated rats (Figures 4(f) and 4(g)).

### 3.4. The Localization of Glut2 Was Normalized After DJB

**Figure 5:**
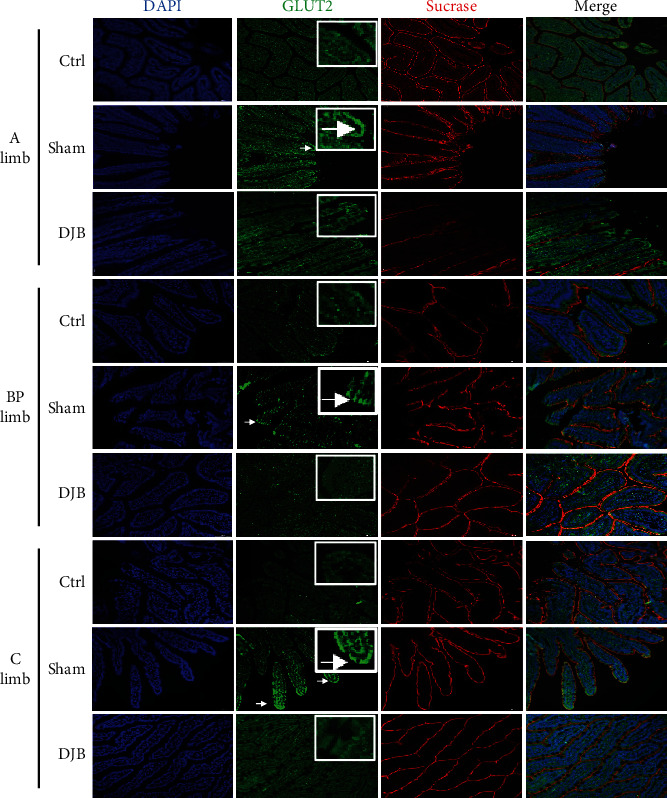
GLUT2 localization was normalized in the small intestine after DJB. Immunostaining of GLUT2 (green) and sucrase (red) in the apical membranes of intestinal epithelial cells and DAPI in the nucleus (blue) in sections of rat intestinal alimentary limbs. White arrows indicate the localization of GLUT2 in the apical membranes of the intestinal epithelial cells in the T2D-Sham groups.

In addition to the important role of glucose sensing in glycemic control, the localization of GLUT2 is also closely related to glucose absorption in the small intestine. To identify the changes of GLUT2 in DJB-improved glucose control, we detected the expression of GLUT2 in the A, BP, and C limbs by immunofluorescence under fasting condition. The immunostaining results showed that GLUT2 was colocalized with sucrase in the apical membranes of the proximal intestinal epithelial cells of T2D-Sham rats, which were located in the basolateral membranes in the Ctrl rats (Figure 5). However, GLUT2 localization was normalized in the basolateral membranes after DJB (Figure 5).

### 3.5. DJB-Improved Glucose Tolerance Was Reversed by the Inhibition of STRs

**Figure 6:**
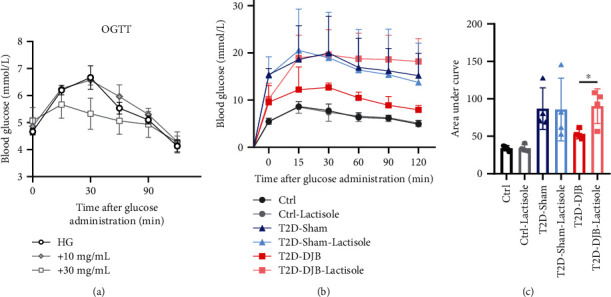
The effect of DJB-improved glycemic control was inhibited by lactisole. (a) The dose of lactisole was tested in control rats. OGTT results after lactisole administration with (b) high glucose and the (c) area under curve as indicated. ^∗^*p* < 0.05.

In Figure 4, our results showed that the expression of STRs in the three intestinal segments was obviously altered after DJB operation, and we used the STR inhibitor lactisole to verify the role of STRs in DJB-improved glucose control in vivo for the first time. The dose of lactisole was tested in control rats (Figure 6(a)), and a relatively low dose of lactisole was administered during the OGTT after DJB. The results showed that the DJB-improved restoration of glucose tolerance was cancelled by lactisole administration without significant effects in the Ctrl and sham groups (Figures 6(b) and 6(c)).

## 4. Discussion

Bariatric surgery has been one of the most effective therapeutic approaches in the management of obesity and diabetes in both humans [2, 13] and rodents [14, 15]. In this study, DJB surgery was performed in an STZ-induced diabetic rat model to identify the mechanisms of DJB-induced effects in the small intestine. Our data showed that glycemic control was improved early after DJB without changes in food intake and body weight, and the plasma levels of insulin, GLP-1, and GLP-2 in DJB-operated rats were increased. We identified adaptive changes in villus height and crypt depth and alterations in glucose sensing signals (T1R2/T1R3, G*α*-gustducin) and glucose transporters (SGLT1, GLUT2) in the A limb, BP limb, and C limb after DJB. The in vivo results showed that the DJB-improved restoration of glucose tolerance was reversed by administration of a moderate dose of lactisole. Our studies indicate that DJB ameliorates glucose control probably by restoring glucose sensing and absorption with the coordination of GLP-1 and GLP-2 in the small intestine.

It is well known that GLP-1 is released from enteroendocrine L cells, which could stimulate insulin release dependent on gut lumen glucose concentrations [13]. Many studies have revealed that GLP-1 is involved in glycemic improvement after bariatric surgery [10, 16, 17]. Our study found that the blood levels of GLP-1 were elevated in DJB-operated diabetic rats under normal chow diet feeding conditions but not in fasting conditions, consistent with previous reports [3, 18]. In addition, two recent studies demonstrated that GLP-1 levels after DJB were increased after glucose administration in a rat model of Type 1 diabetes [19] and Type 2 diabetes [20]. However, the role of GLP-2, which is coreleased with GLP-1 from L cells, remains largely unclear in bariatric surgery–improved glycemic control. In humans, circulating GLP-2 concentrations were found to be increased after RYGB [21], which might be correlated with satiety regulation by RYGB [22]. In rodents, for the first time, we examined the changes in both GLP-1 and GLP-2 levels under feeding and fasting conditions after DJB. Our data showed that the circulating concentrations of GLP-2 were also elevated in DJB-operated diabetic rats compared to the sham group. Yang et al. confirmed the finding that the fasting blood GLP-2 level of DJB rats was higher than that of sham rats [23]. GLP-2 has been reported to stimulate gut mucosa cell proliferation [24, 25] and induce intestinal expansion [23, 26, 27], which supports our results of DJB-modulated hypertrophy of the A limb. Taken together, these findings suggest that the responses of GLP-1 and GLP-2 to luminal glucose levels are involved in DJB-induced intestinal adaptation and glycemic improvements. Smith et al. found that GLP-1 and GLP-2 were coordinated in regulating glucose homeostasis, whereas GLP-1 induced insulin secretion and GLP-2 caused GLUT2 translocation to complement SGLT1-mediated glucose absorption in response to a meal rich in sugars [7]. Markedly, they demonstrated that the coordination of GLP-1 and GLP-2 needed T1R2-mediated glucose sensing. Another group reported that the responses of GLP-1 were reduced in T1R3-deficient mice or taste G*α*-gust-deficient mice [28]. These findings indicated a link between intestinal glucose sensing and glycemic control.

Lactisole, an STR inhibitor for humans and other primates, was found to reduce the release of GLP-1 during OGTT with mildly increased glucose levels in healthy humans [29]. In addition, lactisole also inhibited GLP-1 secretion in rodent enteroendocrine L cells STC-1 by downregulating STR expression [30, 31]. Consistently, our in vivo results showed that DJB-improved glycemic control was inhibited with lactisole administration, probably by downregulating STRs. There were no effects on glucose tolerance in the control and sham groups with lactisole administration, possibly because of the moderate dose of lactisole used in the study.

In health, glucose is primarily absorbed by SGLT1 and transported to the circulation via GLUT2, subsequently triggering the release of GLP-1 and GLP-2 [6, 32] during a normal meal. In diabetes, the expression of SGLT1 and GLUT2 in the intestine is increased [33], while the expression of T1R2/T1R3 is decreased with reduced GLP-1 and GLP-2 levels, indicating the dysregulation of intestinal taste signaling [34]. In addition, it was found that the activity of SGLT1 was mediated by T1R2/T1R3 [6, 35] and that T1R2/T1R3 activation stimulated GLP-1/GLP-2 release [7]. In this study, the expression of glucose sensing signals (T1R2/T1R3, G*α*-gust) and glucose transporters (SGLT1, GLUT2) was decreased in the BP limb and C limb, but T1R2, G*α*-gust, and SGLT1 expression levels were upregulated while GLUT2 was downregulated in the A limb of DJB-operated rats fed a chow diet. These previous studies supported our opinion that DJB-induced glycemic improvement probably occurs by restoring T1R2 signaling and SGLT1 function with the responses of GLP-1 and GLP-2 to a meal. Moreover, our RNA-Seq data analysis revealed that glucose metabolism was improved in the A limb after DJB (Figure S1), consistent with our study [12] and previous reports [12, 36]. Furthermore, our results of T1R2 and SGLT1 upregulation in the A limb after DJB were consistent with the report by Kim et al. [37] but contrary to the study by Jurowich et al. [14], perhaps due to the difference in the samples that were collected under feeding or fasting. The roles of T1R2 and SGLT1 need to be further investigated in metabolic surgery.

A clinical limitation is that DJB surgery improves glycemic control without weight loss compared with SG or RYGB bariatric surgeries. In addition to GLP-1, GLP-2, STR, SGLT1, and GLUT2 which was involved in DJB-ameliorated glucose metabolism, our RNA sequencing data also showed that the expression of ghrelin and growth hormone secretagogue receptor (Ghsr) was altered in the DJB group compared with the sham group. Combined with our previous study in the brain tissue [4], DJB may improve glucose metabolism systemically such as via the gut-brain axis. Therefore, the effects of DJB on T2D warrant further investigation.

## 5. Conclusion

This study demonstrated that DJB improved the levels of insulin, GLP-1, and GLP-2 and altered the expression of STR, SGLT1, and GLUT2 in each limb of the intestine. Additionally, we found that the localization of GLUT2 was restored in the basolateral membranes of intestinal epithelial cells after DJB which was in the apical membranes of intestinal epithelial cells in T2D-sham rats. Furthermore, our results showed that DJB-ameliorated glycemic control was suppressed by STR inhibitor lactisole. In summary, our research indicated that DJB improved diabetic glucose metabolism probably by repairing T1R2/T1R3-mediated glucose sensing and SGLT1/GLUT2-regulated glucose absorption with the coordination of gastrointestinal hormones.

## Data Availability

The datasets used and/or analyzed that support the findings of this study are available from the corresponding authors upon reasonable request.

## Supporting Information

Supporting Information 1 Table S1. Primers used in this study.

Supporting Information 2 Figure S1. Glucose metabolism was improved in the alimentary limb after DJB.
