# Supplementary material for: Duodenal–Jejunal Bypass Restores Sweet Taste Receptor-Mediated Glucose Sensing and Absorption in Diabetic Rats
Source: J Diabetes Res. 2024 Sep 4;2024:5544296. doi: 10.1155/2024/5544296 (PMC11390237; doi:10.1155/2024/5544296)
Supplement: Supporting Information 1 — Table S1. Primers used in this study. [file 5544296.f1.docx]

Supplementary Table 1. Primers used in this study

| Gene Primer | Sequences | |
| --- | --- | --- |
| T1r2 forward | 5’TTCTCATGCTTCTGCCGACAG3’ |  |
| T1r2 reverse | 3’GCCATCTTGAAGACACACACGA5’ |  |
| T1r3 forward | 5’ATGTAGTGGCCAGGCAACC3’ |  |
| T1r3 reverse | 3’ACC TGGCCATCTTTGCACT5’ |  |
| Gα-gustducin forward | 5’GCGATCCAGGAATTCAAGCCT3’ |  |
| Gα-gustducin reverse | 3’GATACCAGTGGTTTTCACCCGG5’ |  |
| Sglt1 forward | 5’ATTGGAATCTCCCGTATG3’ |  |
| Sglt1 reverse | 3’ATGACGAAGAGGATGATG5’ |  |
| Glut2 forward | 5’CCAGCACATACGACACCAGACG3’ |  |
| Glut2 reverse  Gcg forward  Gcg reverse | 3’CCAAAGAACGAGGCGACCAT5’  5’CGGAAGAAGTCGCCATAGCTGAG3’  3’TTGATGAAGTCTCTGGTGGCAAGG5’ |  |
| Gapdh forward | 5’GCATGGCCTTCCGTGTTCCTA3’ |  |
| Gapdh reverse | 3’GATGCCTGCTTCACCACCTTCT5’ |  |

Supplementary Figure 1 Glucose metabolism was improved in alimentary limb after DJB. GSEA enrichment analysis showed the reactome of glucose metabolism (a), glycolysis (b), gluconeogenesis (c) and TCA cycle (d). NES=Normalized Enrichment Score; positive and negative values of NES indicated up- and downregulated gene expression patterns, respectively.
