# Supplementary figures and images for: Duodenal–Jejunal Bypass Restores Sweet Taste Receptor-Mediated Glucose Sensing and Absorption in Diabetic Rats
Source: J Diabetes Res. 2024 Sep 4;2024:5544296. doi: 10.1155/2024/5544296 (PMC11390237; doi:10.1155/2024/5544296)

(a)

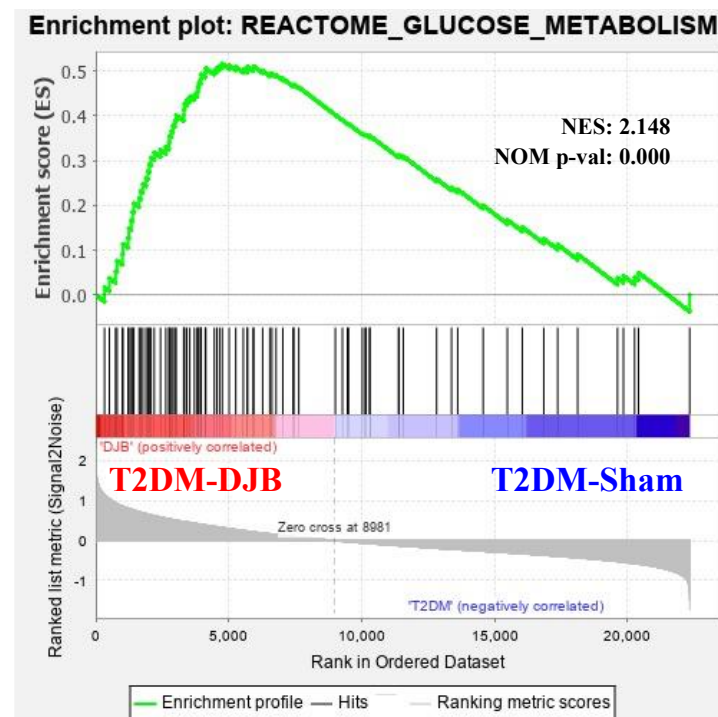

(b)

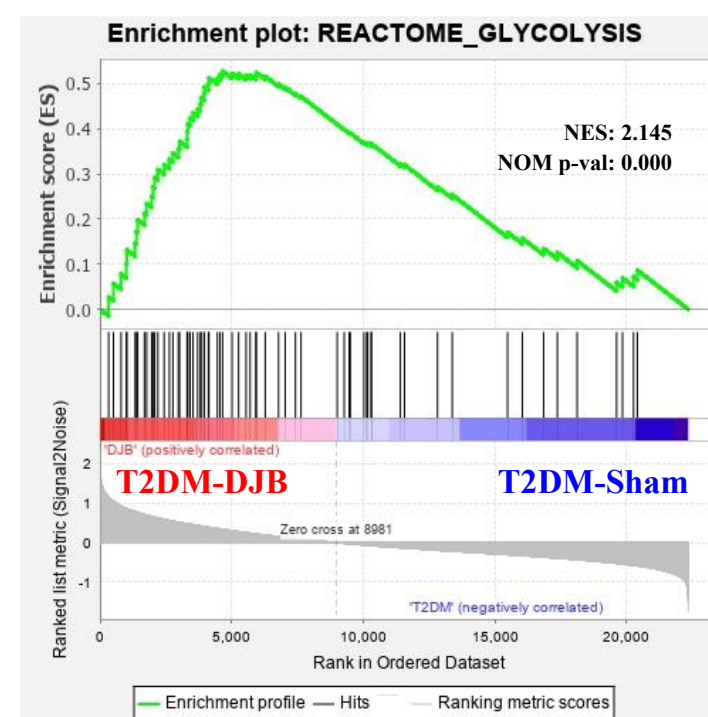

(c)

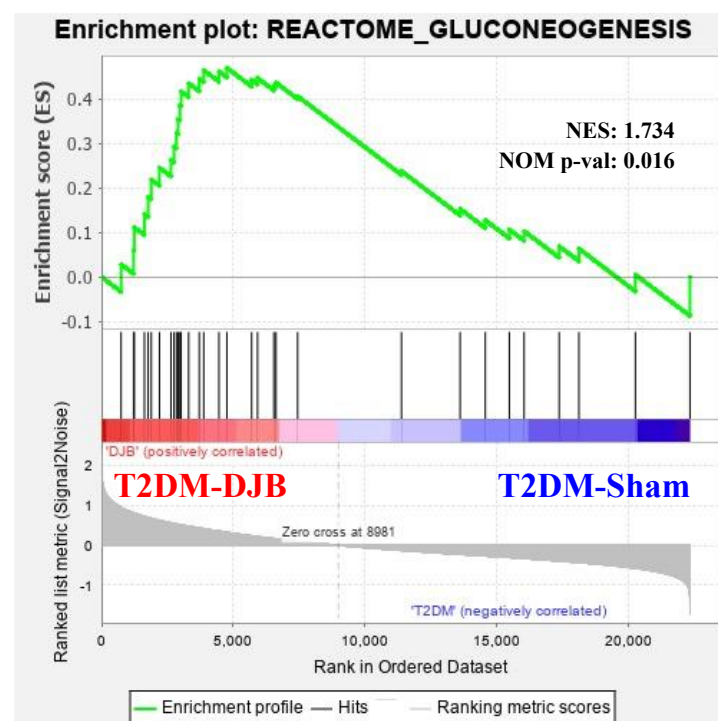

(d)

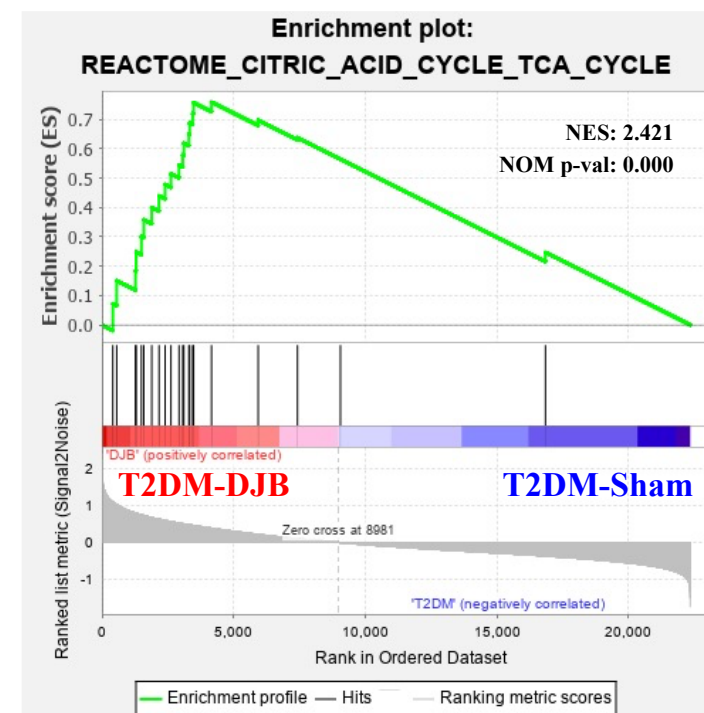

Supplement: Supporting Information 2 — Figure S1. Glucose metabolism was improved in the alimentary limb after DJB. [file 5544296.f2.pdf]
